# Molecular insight into 2-phosphoglycolate activation of the phosphatase activity of bisphosphoglycerate mutase

**DOI:** 10.1107/S2059798322001802

**Published:** 2022-03-11

**Authors:** Anfal S. Aljahdali, Faik N. Musayev, John W. Burgner, Mohini S. Ghatge, Vibha Shekar, Yan Zhang, Abdelsattar M. Omar, Martin K. Safo

**Affiliations:** a Department of Pharmaceutical Chemistry, King Abdulaziz University, Alsulaymanyah, Jeddah 21589, Saudi Arabia; b Department of Medicinal Chemistry, School of Pharmacy, Virginia Commonwealth University, 800 East Leigh Street, Richmond, VA 23298, USA; c The Institute for Structural Biology, Drug Discovery and Development, Virginia Commonwealth University, 800 East Leigh Street, Richmond, VA 23298, USA; d Department of Pharmaceutical Chemistry, Al-Azhar University, Cairo 11884, Egypt

**Keywords:** bisphosphoglycerate mutase, 2,3-bisphosphoglycerate, 2-phosphoglycolate, synthases, phosphatases, kinetics, X-ray crystallography

## Abstract

Bisphosphoglycerate mutase (BPGM) is an erythrocyte-specific multifunctional enzyme that is responsible for the regulation of 2,3-bisphosphoglycerate (2,3-BPG) in red blood cells through its synthase and phosphatase activities; the latter enzymatic function is stimulated by the endogenous activator 2-phosphoglycolate (2-PG). 2,3-BPG is a natural allosteric effector of hemoglobin (Hb) that is responsible for decreasing the affinity of Hb for oxygen to facilitate tissue oxygenation. Here, crystal structures of BPGM with 2-PG in the presence and absence of 3-phosphoglycerate are reported at 2.25 and 2.48 Å resolution, respectively. Structure analysis revealed a new binding site for 2-PG at the dimer interface for the first time, in addition to the expected active-site binding. Also, conformational non-equivalence of the two active sites was observed as one of the sites was found in an open conformation, with the residues at the active-site entrance, including Arg100, Arg116 and Arg117, and the C-terminus disordered. The kinetic result is consistent with the binding of 2-PG to an allosteric or noncatalytic site as well as the active site. This study paves the way for the rational targeting of BPGM for therapeutic purposes, especially for the treatment of sickle cell disease.

## Abstract 2

Crystal structures of bisphosphoglycerate mutase (BPGM) with 2-phosphoglycolate in the presence and absence of 2,3-bisphosphoglycerate are reported. The structures identified a novel binding site for 2-phosphoglycolate at the dimer interface of BPGM, as well as showing a snapshot of the catalytic activity of BPGM.

## 1. Introduction

Bisphosphoglycerate mutase (BPGM) is a central enzyme in the Rapoport–Luebering shunt, a side glycolytic pathway that is present predominantly in the mammalian erythrocyte (Rapoport & Luebering, 1950 ▸, 1951 ▸; Rose, 1968 ▸). BPGM is a multifunctional enzyme, functioning as a 2,3-BPG synthase, a 2,3-BPG phosphatase and a phosphoglycerate mutase. All three activities occur at the same site, with the synthase activity being the main activity of the enzyme. BPGM synthase catalyzes the isomerization of the glycolytic intermediate 1,3-bisphosphoglycerate (1,3-BPG) to 2,3-BPG, which is an allosteric effector of hemoglobin (Hb) (Rose & Whalen, 1973 ▸; Rose & Dube, 1976 ▸; Ikura *et al.*, 1976 ▸). The phosphatase activity is responsible for the hydrolysis of 2,3-BPG to 3-phosphoglycerate (3-PGA), an intermediate in the main glycolytic pathway. The phosphatase activity is inherently low (approximately 1000-fold lower than the synthase activity) and has been reported to be stimulated by several anions, such as chloride, phosphate, sulfite and, most potently, 2-phospho­glycolate (2-PG) (Rose & Liebowitz, 1970 ▸; Reynolds, 1986 ▸; Yu *et al.*, 1990 ▸). In addition, BPGM exhibits a phosphoglycerate mutase activity similar to the main activity of the glycolytic enzyme monophosphoglycerate mutase (PGAM), which catalyzes the conversion of 3-PGA to 2-phosphoglycerate (2-PGA) in the main glycolysis (Rose, 1968 ▸). However, the mutase activity of BPGM has been reported to be physio­logically insignificant (Sasaki & Chiba, 1983 ▸).

The regulation of 2,3-BPG levels in erythrocytes by the activity of BPGM is crucial in regulating the allosteric activity of Hb and consequently tissue oxygenation. During deoxy­genation of erythrocytes, low oxygen tension activates glycol­ysis, which in turn increases the concentration of 2,3-BPG (Sun *et al.*, 2016 ▸; Zhang *et al.*, 2014 ▸). 2,3-BPG preferentially binds and stabilizes deoxygenated Hb, allowing oxygen to be released to tissues in need (Benesch & Benesch, 1967 ▸, 1969 ▸; Chanutin & Curnish, 1967 ▸). This adaptation mechanism influences the efficiency of oxygen dissociation from Hb and optimizes oxygen delivery to tissues. However, in clinical conditions such as sickle cell disease (SCD), where oxygen transport is consistently compromised, 2,3-BPG increases to an abnormally high concentration, inducing the primary pathophysiological events of the disease.

SCD is a hereditary hematological disorder characterized by dysfunctional sickle Hb (HbS) caused by a single point mutation that replaces glutamic acid at the sixth position of the Hb β-globin gene with valine (β6 Glu→Val; Bunn, 1997 ▸; Steinberg, 2008 ▸). Under normal oxygen saturation levels both Hb and HbS have an identical structure (Bunn, 1997 ▸; Steinberg, 2008 ▸). However, during a state of hypoxia, dissociation of oxygen from HbS exposes the mutated βVal6 to a complementary hydrophobic site on the surface of an adjacent deoxygenated HbS, associating the molecules together and consequently forming polymers that convert the normal and flexible biconcave red blood cells (RBC) into rigid sickle-shaped RBC (Harrington *et al.*, 1997 ▸; Ghatge *et al.*, 2016 ▸; Bunn & Forget, 1986 ▸; Eaton & Hofrichter, 1990 ▸). This hypoxia-induced HbS polymerization is the hallmark of SCD pathology, and the elevated 2,3-BPG in sickle RBC significantly contributes to this pathophysiology by decreasing the affinity of HbS for oxygen and increasing the concentration of deoxygenated HbS, consequently exacerbating HbS polymerization and RBC sickling (Poillon *et al.*, 1985 ▸, 1995 ▸).

The reduction of intracellular levels of 2,3-BPG by activating BPGM phosphatase with the activator 2-PG has been investigated by Poillon and coworkers and has been shown to improve the solubility of HbS and prevent the sickling tendency of RBC (Poillon *et al.*, 1985 ▸, 1995 ▸). A recent study reported that an SCD mouse model lacking the BPGM gene showed an improvement in the concentration of the non­polymerizing oxygenated HbS and RBC sickling (Knee *et al.*, 2020 ▸). However, despite the accumulated knowledge about the involvement of BPGM in SCD pathology and BPGM phosphatase activation by 2-PG, the molecular mechanism of 2-PG activation is poorly understood. In addition, targeting BPGM for therapeutic purposes has yet to be investigated, in part due to its complex kinetic mechanism.

The BPGM phosphatase mechanism has been elucidated in most part from crystal structures of BPGM in complex with 2,3-BPG (PDB entries 2hhj and 2h4z) and shows an S_N_2 mechanism in which His11 at the active site of BPGM attacks 2,3-BPG to produce a phosphoenzyme catalytic intermediate and 3-PGA as a product (Wang *et al.*, 2006 ▸). Subsequently, 3-PGA dissociates from the active site, allowing an activated water molecule to act as a second substrate, hydrolyzing the phosphoenzyme to restore the free enzyme form (Rose & Liebowitz, 1970 ▸; Wang *et al.*, 2006 ▸). It is noteworthy that the mechanism of activation by 2-PG has been postulated to occur at the active site of BPGM, in which 2-PG acts as a second substrate. In contrast, unlike 3-PGA, 2-PG cannot accept a phosphoryl group, facilitating the transfer of the enzyme-bound phosphoryl group to a water molecule (Rose, 1980 ▸). However, the exact binding mode and activation mechanism of 2-PG are only speculative and have not been fully elucidated.

The present paper aims to elucidate the atomic interaction of BPGM with 2-PG in the presence and absence of 2,3-BPG and the kinetic characterization of the 2-PG activation mechanism. The findings are expected to shed new light on the activation of the BPGM phosphatase mechanism and to provide valuable insights into, and foundations for, the design of phosphatase activators with potential therapeutic benefits, including in SCD.

## 2. Experimental

### 2.1. BPGM expression and purification

A human BPGM clone in pET-30b(+) vector was purchased from GenScript and transformed into *Escherichia coli* BL21-CodonPlus (DE3)-RIPL cells for expression. The transformed cells were streaked on a kanamycin plate and incubated overnight at 37°C. A single colony was picked from the kanamycin plate and grown overnight with shaking at 37°C in Luria–Bertani (LB) broth containing 100 µg ml ^−1^ kanamycin. The culture was then inoculated (1:20) into 6 l LB medium and grown at 37°C under aerobic conditions until the optical density at 600 nm (OD_600_) reached 0.6. Next, the cell culture was induced with 0.2 m*M* isopropyl β-d-1-thiogalactopyranosidase (IPTG) and further incubated at 30°C for 8 h. The cells were harvested by centrifugation and the cell pellets were resuspended in a buffer consisting of 50 m*M* Tris–HCl, 10 m*M* imidazole, 300 m*M* NaCl pH 8 (buffer *A*). The resuspended cells were lysed using an Avestin Emulsiflex (Emulsiflex C-3, operating at >137 MPa), followed by high-speed centrifugation at 12 000 rev min^−1^ for 20 min. The supernatant was filtered and loaded onto a 5 ml HisTrap HP column (GE Healthcare Bio-Sciences) pre-equilibrated with buffer *A*.

The purification process was carried out using ÄKTA fast protein liquid chromatography (FPLC). First, the column was washed with buffer *A* containing a low concentration of imidazole (10 m*M*) to remove nonspecific binding proteins. An imidazole gradient was then used with a buffer consisting of 50 m*M* Tris–HCl, 250 m*M* imidazole, 300 m*M* NaCl pH 8 (buffer *B*); the protein eluted at 75 m*M* imidazole. Protein fractions were collected and their purity was assessed by the presence of a single band on sodium dodecyl sulfate polyacrylamide gel electrophoresis (SDS–PAGE). The pure fractions were pooled and dialyzed overnight at 4°C against a buffer consisting of 20 m*M* Tris–HCl, 150 m*M* NaCl pH 7.5. The dialysis buffer was then exchanged to a second dialysis buffer containing a lower salt concentration (20 m*M* Tris–HCl, 100 m*M* NaCl pH 7.5) and dialyzed for 4 h. The protein concentration was determined spectrophotometrically using an extinction coefficient at 280 nm of 1.63 a.u. = 1 mg ml^−1^.

### 2.2. Sedimentation-velocity analytical ultracentrifugation experiment

A sedimentation-velocity (SV) experiment using absorbance and interference detectors was carried out in a Proteome Lab XL-I analytical ultracentrifuge (AUC; Beckman Coulter, Indianapolis, Indiana, USA) following the standard protocols (Zhao *et al.*, 2013 ▸). A tenfold concentration range, 5–50 µg ml^−1^, of purified BPGM was used. The experimental temperature was 20°C. The samples were loaded into AUC cell assemblies with 12 mm charcoal-filled Epon double-sector centerpieces and sapphire windows. The sample cells were loaded into a rotor for temperature equilibration for 1.5 h under vacuum in the AUC followed by acceleration to full speed at 42 000 rev min^−1^. Continuous radial scans were initiated immediately using the selected detection systems. The accuracy of the determined *s* values is ∼1% or better (Zhao *et al.*, 2015 ▸; Ghirlando *et al.*, 2013 ▸). The resulting SV data were analyzed using the continuous *c*(*s*) module in *SEDFIT* version 16.36 as described in Schuck *et al.* (2016 ▸)

### 2.3. Structure determination of BPGM in complex with 2-PG in the presence and absence of 3-PGA

Purified BPGM (30 mg ml^−1^) in 20 m*M* Tris–HCl, 100 m*M* NaCl pH 7.5 was incubated with 3.8 m*M* 2,3-BPG and 7.6 m*M* 2-PG for ternary complex formation and with 8.3 m*M* 2-PG for binary complex formation at room temperature for 1 h. Crystallization of the complexes was carried out by the sitting-drop vapor-diffusion method using a Crystal Gryphon robot (Art Robbins Instruments) by screening against the PEGRx HT crystallization screen (Hampton Research). X-ray quality crystals were obtained in about 15 days using a condition consisting of 0.1 *M* bis-Tris propane, 18% polyethylene glycol 8000, 10%(*v*/*v*) polyethylene glycol 200. For both crystallization experiments, crystals were mounted for X-ray data collection after being flash-cooled in liquid nitrogen. The crystallization condition required no cryoprotectant. X-ray diffraction data were collected at 100 K using a Rigaku MicroMax-007 HF X-ray generator and an EIGER R 4M detector. The data set was processed with *CrysAlis^Pro^* 40.64.69a (Rigaku) and the *CCP*4 suite of programs (Winn *et al.*, 2011 ▸).

The crystal structure of the ternary complex was solved with the *Phaser-MR* (simple interface) molecular-replacement program in the *Phenix* software package (Liebschner *et al.*, 2019 ▸) using the monomeric crystal structure of BPGM in complex with 2,3-BPG (PDB entry 2h4z) as the search model (Wang *et al.*, 2006 ▸). The structure was refined with the *Phenix* software along with model building using the graphics program *Coot* (Liebschner *et al.*, 2019 ▸; Emsley *et al.*, 2010 ▸; Afonine *et al.*, 2012 ▸). The refined ternary BPGM complex without bound ligands and water molecules was used as the starting model for refinement of the diffraction data for the binary BPGM complex.

### 2.4. Kinetic studies of the 2-PG activation mechanism

The kinetic study was performed using the method described by Calvin *et al.* (1990 ▸). The reaction was carried out in a final volume of 200 µl in clear flat-bottomed 96-well plates using a BMG Labtech CLARIOstar Plus microplate reader. The standard assay mixture consisted of 50 m*M* Tris–HCl buffer pH 7.5, 10 m*M* magnesium chloride, 0.2 m*M* reduced nicotinamide adenine dinucleotide (NADH), 3 m*M* adenosine triphosphate (ATP), 3.3 U ml^−1^ glyceraldehyde 3-phosphate dehydrogenase (GAPDH), 2.3 U ml^−1^ phospho­glycerate kinase (PGK), varying concentrations of 2,3-BPG (3–2000 µ*M*) and four different fixed 2-PG concentrations (10, 50, 250 and 500 µ*M*). The reaction was initiated by the addition of 2 µ*M* BPGM and was monitored for 20 min. The initial velocity (Δ*A*_340 nm_ min^−1^) was calculated from the slope of the tangent line in the reaction-progress curve. The absorbance was then converted into micromoles of NADH per minute using the millimolar NADH extinction coefficient (ɛ_340_ = 6.22 cm^−1^ m*M* ^−1^). The activity curve was obtained by plotting the initial velocity (micromoles of NADH per minute) versus the BPGM concentration using *Microsoft Excel*. The initial velocity at each 2,3-BPG concentration with a fixed 2-PG concentration was plotted against the 2,3-BPG concentration using the Michaelis–Menten nonlinear regression model in *GraphPad Prism* 8 to obtain *K*_m_, *V*_max_ and *k*_cat_ values.

## 3. Results

### 3.1. Crystallographic study of BPGM in complex with 3-PGA and 2-PG

**Figure 1:**
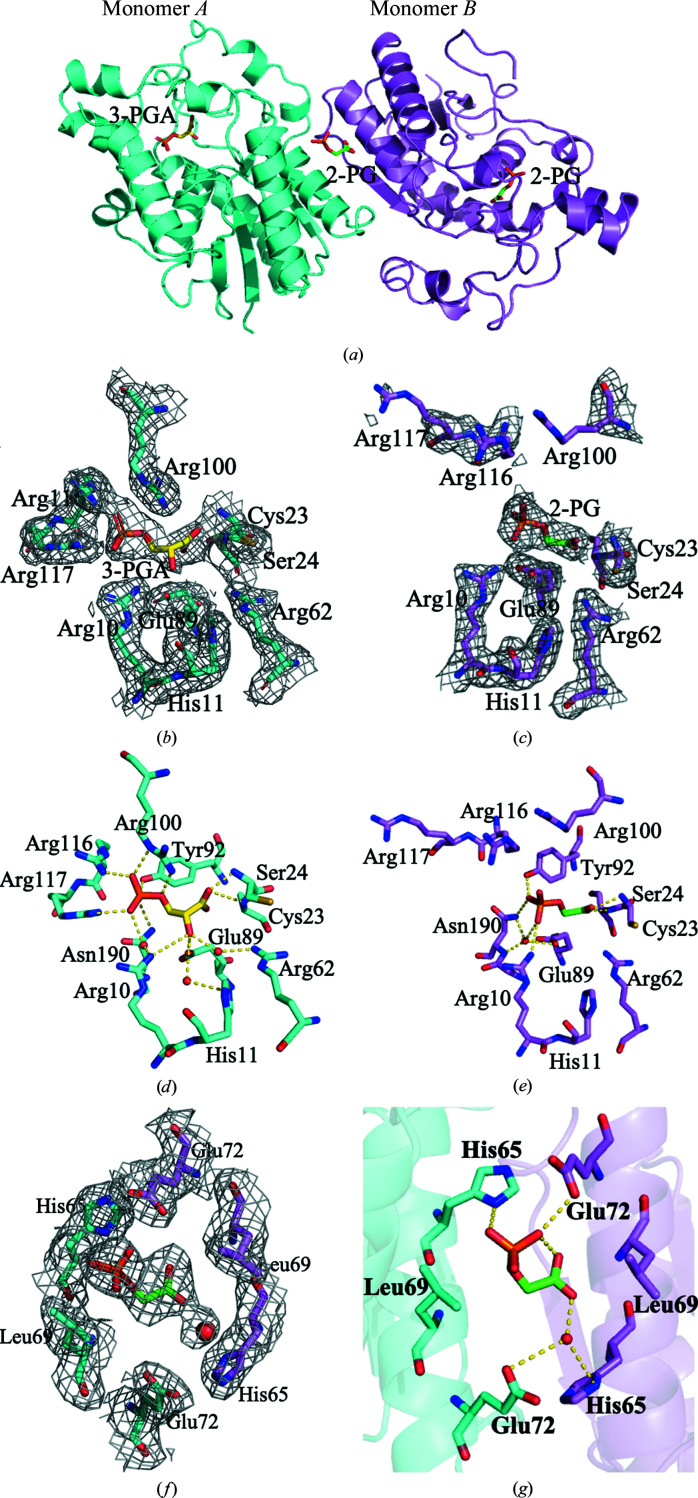
Crystal structure of the ternary BPGM·3-PGA·2-PG complex. (*a*) Overall structure of BPGM·3-PGA·2-PG (ribbons) with 3-PGA (sticks) bound at the active site of monomer *A* and 2-­PG (sticks) bound at the active site of monomer *B* and at the dimer interface. (*b*) Electron-density map of the active site of monomer *A* with bound 3-PGA (2*F*_o_ − *F*_c_ map contoured at the 0.8σ level). (*c*) Electron-density map of the active site of monomer *B* with bound 2-PG (2*F*_o_ − *F*_c_ map contoured at the 0.8σ level). (*d*) Detailed interactions between the active-site residues of monomer *A* (cyan sticks) and 3-PGA (yellow sticks). (*e*) Detailed interactions between the active-site residues of monomer *B* (pink sticks) and 2-PG (green sticks). (*f*) Electron-density map of the dimer interface residues with bound 2-PG (2*F*_o_ − *F*_c_ map contoured at the 0.8σ level). (*g*) Detailed interactions between the dimer interface residues (cyan sticks for monomer *A* and pink sticks for monomer *B*) and 2-PG (green sticks).

The crystal structure of BPGM complexed with 3-PGA and 2-PG has been determined to 2.25 Å resolution. The data-collection and refinement statistics are summarized in Table 1 ▸. The crystal structure is isomorphous to PDB entry 2h4z. The asymmetric unit contains two monomers, *A* and *B*, that are associated together by twofold noncrystallographic symmetry (Fig. 1 ▸ *a*). The two monomers appear to form a homodimer in solution, as suggested by an analytical centrifugation (AUC) experiment. Analysis of the data from the SV experiment indicates that BPGM does not dissociate under the conditions chosen for the experiment. This conclusion is based on the observation that the sedimentation coefficient, *S*_20,w_, for the predominant species in each of the SV experiments only increases slightly, rather than decreases as would be expected for a dimer that does dissociate in the concentration range used here (Supplementary Fig. S1). In fact, *S*_20,w_ increases only slightly with decreasing BPGM concentration, as would be expected if dissociation were not occurring. If dissociation is occurring, it does so extremely slowly and its *K*_d_ must be in the range of at least 10^−12^ *M*. In addition, the experimentally determined values for the physical properties of BPGM as well as those calculated by *HYDROPRO*10 (Supplementary Table S1) show that the observed *S*_20,w_ as a function of concentration is more consistent with a stable dimer.

The monomeric BPGM protein consists of 259 amino acids. BPGM has two active sites in the C-terminal portion of an α/β domain of each monomer. The overall dimeric structure is identical to the previously published liganded and unliganded BPGM structures (PDB entries 1t8p, 2h4x, 2h4z, 2hhj and 3nfy; Wang *et al.*, 2004 ▸, 2006 ▸; Patterson *et al.*, 2010 ▸).

Even though BPGM was co-crystallized with 2,3-BPG and 2-PG, the electron density at the active site of monomer *A* suggested bound 3-PGA, which is a product of the hydrolysis of 2,3-BPG (Figs. 1 ▸ *a* and 1 ▸ *b*) by the phosphatase activity of BPGM, which might have occurred during the incubation/crystallization process. Interestingly, the effector 2-PG was found bound to the active site of monomer *B* (Figs. 1 ▸ *a* and 1 ▸ *c*), as well as to the dimer interface, close to the twofold non­crystallographic axis (Figs. 1 ▸ *a* and 1 ▸ *e*). The *B* factor of 3-PGA in monomer *A* is 39.0 Å^2^, that of 2-PG in monomer *B* is 63.9 Å^2^ and that of 2-PG in the dimer interface is 56.3 Å^2^, consistent with the weakest ligand density being observed in monomer *B*, and likely suggesting different affinities for the ligands at these binding sites. This ternary complex structure will be referred to as BPGM·3-PGA·2-PG.

In monomer *A* with bound 3-PGA, we observed electron density for residues Ser2–Val254, similar to the published structures of BPGM in complex with 2,3-BPG (PDB entry 2h4z) and 3-PGA (PDB entry 2h4x). However, unlike monomer *A*, a significant portion of the C-terminus of monomer *B*, which partly forms the active-site structure, was missing (13 residues) due to disorder. Also, unlike monomer *A*, the side chains of several other amino acids located in the active-site pocket of monomer *B*, including Arg100, Arg116 and Arg117, were highly disordered, as shown by weak or missing electron density (Figs. 1 ▸ *b* and 1 ▸ *c*). The disorder at the active site of monomer *B* has led to what appears to be an open active-site conformation, even though there is a bound 2-PG (Fig. 1 ▸ *c*). It is notable that similar disorder and an open active-site conformation are observed in the unliganded structure of BPGM (PDB entry 3nfy; Patterson *et al.*, 2010 ▸). In contrast, monomer *A* with bound 3-PGA shows a closed conformation, as the C-terminus and the residues Arg100, Arg116 and Arg117 that partly form the active site are well ordered and are involved in several interactions (Fig. 1 ▸ *b*). A similar well ordered and closed active-site conformation was observed for the previously reported structures of BPGM in complex with 2,3-BPG (PDB entry 2h4z) or 3-PGA (PDB entry 2h4x) (Wang *et al.*, 2006 ▸). It appears that the conformational behavior of the C-terminus and the side chains of Arg100, Arg116 and Arg117 lead to a structural transition between open and closed active-site conformations.

The molecular interactions of 3-PGA at the active site of monomer *A* (Fig. 1 ▸ *d* and Supplementary Fig. S2*a* ) are similar to the previously reported structure of BPGM in complex with 3-PGA (PDB entry 2h4x; Wang *et al.*, 2006 ▸). The 3-PGA phosphoryl O atoms interact with the guanidino groups of Arg100 (2.7–2.9 Å), Arg116 (2.7 Å) and Arg117 (2.9 Å), the amide of Asn190 (3.2 Å) and the hydroxyl of Tyr92 (2.6 Å). The hydroxyl of 3-PGA makes direct hydrogen-bond interactions with the carboxyl of Glu89 (2.3 Å) and weak inter­actions with the guanidino group of Arg10 (3.6 Å). In addition, the hydroxyl of 3-PGA makes water-mediated interactions with the residues at the bottom of the active site, including His11, Arg62 and Glu89. Finally, the carboxyl of 3-PGA forms hydrogen-bond interactions with the main-chain N atoms of Cys23 (2.3 Å) and Ser24 (3.3 Å).

The 2-PG at the active site of monomer *B* in some instances shows similar binding interactions as described above for 3-PGA (Fig. 1 ▸ *e* and Supplementary Fig. S2*b* ). The 2-PG phosphoryl O atoms form hydrogen-bond interactions with the amide of Asn190 (3.2 Å), the hydroxyl of Tyr92 (2.8 Å) and the guanidino group of Arg10 (3.0 Å). The interactions with the guanidino groups of Arg116 and Arg117 observed in monomer *A* were missing due to disorder of these residues. The carboxyl of 2-PG also makes direct hydrogen-bond interactions with the main-chain N atoms of Cys23 (3.2 Å) and Ser24 (2.4 Å). The carboxyl of 2-PG also makes water-mediated hydrogen-bond interactions with Glu89, Asn190 and Gly189. However, unlike 3-PGA, it does not make any direct hydrogen-bond interactions with Glu89 or water-mediated hydrogen-bond interactions with His11 and Arg62 since only one water molecule was found at the active site of monomer *B*.

As noted above, we also observed a bound 2-PG at the dimer interface that interacts with both monomers *A* and *B* (Figs. 1 ▸ *f* and 1 ▸ *g* and Supplementary Fig. S2*c* ), the first such binding to be reported. The phosphate O atoms form direct hydrogen-bond interactions with the imidazole of His65 (3.3 Å) in monomer *A* and the carboxyl of Glu72 (3.6 Å) in monomer *B*. In addition, the carboxyl group of 2-PG makes water-mediated interactions with the imidazole group of His65 in monomer *B* and the carboxyl of Glu72 in monomer *A*.

In summary, in monomer *A* of the ternary complex the ordered active-site residues Arg100, Arg116, Arg117 and the C-terminal residue Gln251 move towards the bound 3-PGA, which effectively leads to closure of the active-site pocket. By contrast, in monomer *B* these same residues are disordered, as shown by the missing and/or very weak electron density for their side chains, resulting in an open active-site conformation. Clearly, the ligand binds to monomer *A* with higher affinity than to monomer *B*, consistent with the well defined ligand density of the former. The lower *B* factor of the interface ligand relative to the ligand in monomer *B* may also suggest that the former binds with higher affinity than the latter.

### 3.2. Crystallographic study of BPGM in complex with 2-PG

**Figure 2:**
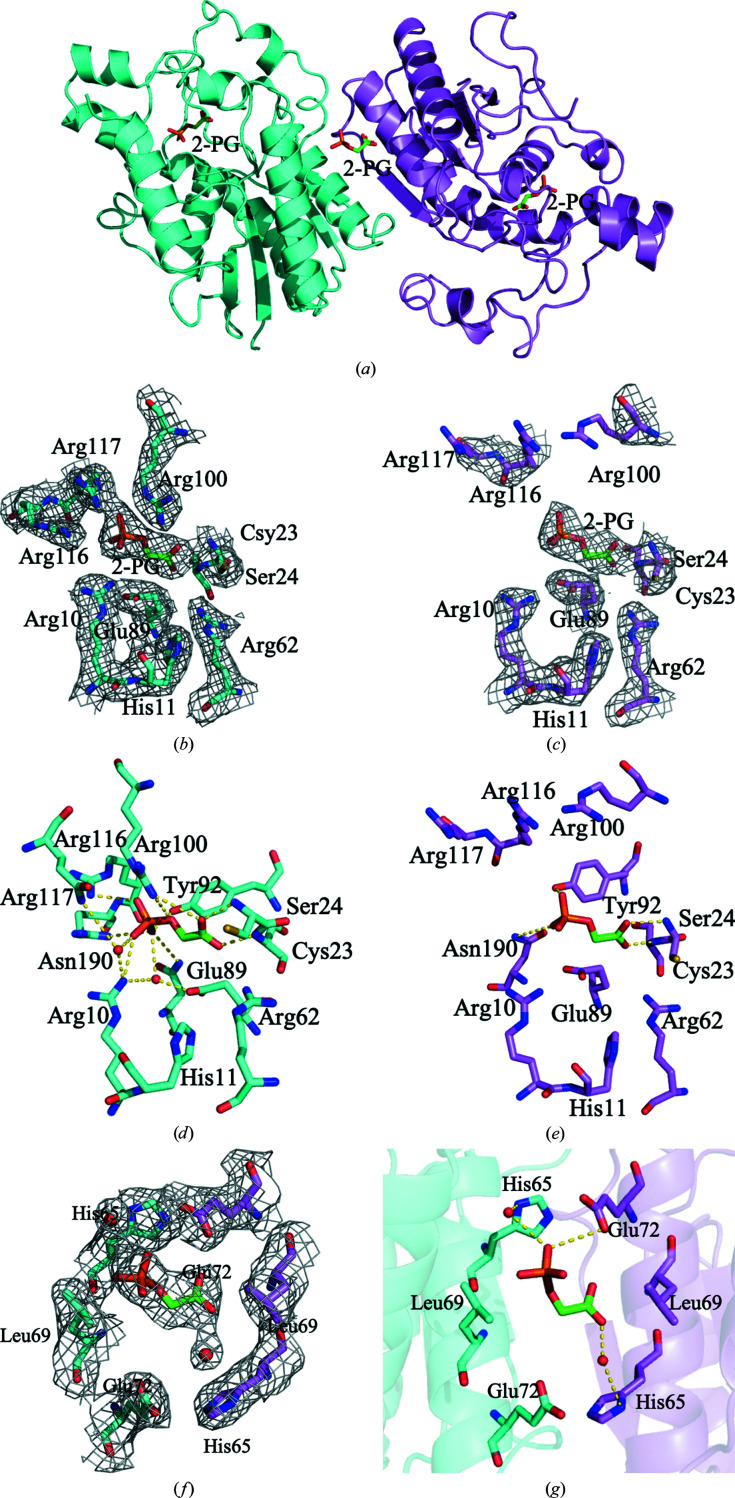
Crystal structure of the binary BPGM·2-PG complex. (*a*) Overall structure of BPGM·2-PG (ribbons) with bound 2-PG (sticks) at the active sites of monomers *A* and *B* and at the dimer interface. (*b*) Electron-density map of the active site of monomer *A* with bound 2-PG (2*F*_o_ − *F*_c_ map contoured at the 0.8σ level). (*c*) Electron-density map of the active site of monomer *B* with bound 2-PG (2*F*_o_ − *F*_c_ map contoured at the 0.8σ level). (*d*) Detailed interactions between the active-site residues of monomer *A* (cyan sticks) and 2-PG (green sticks). (*e*) Detailed interactions between the active-site residues of monomer *B* (pink sticks) and 2-PG (green sticks). (*f*) Electron-density map of the dimer interface with bound 2-PG (2*F*_o_ − *F*_c_ map contoured at the 0.8σ level). (*g*) Detailed interactions between the dimer interface residues (cyan sticks for monomer *A* and pink sticks for monomer *B*) and 2-PG (green sticks).

To confirm the binding mode of 2-PG to BPGM, the crystal structure of BPGM complexed with 2-PG in the absence of 2,3-BPG was also determined to 2.48 Å resolution. The data-collection and refinement statistics are summarized in Table 1 ▸. The binary BPGM·2-PG complex structure showed a 2-PG bound at the active site of both monomers *A* and *B* and at the dimer interface (Fig. 2 ▸ *a*). The overall dimeric structure is identical to that of the ternary BPGM·3-PGA·2-PG structure.

Similar to the ternary BPGM·3-PGA·2-PG complex, the monomer *A* active site of the binary BPGM·2-PG complex was ordered, with continuous electron density from Ser2 to Val254. In contrast, monomer *B* was disordered, with weak or missing electron density at the C-terminus beyond Ile245 and weak or missing side-chain density for Arg100, Arg116 and Arg117. As observed in the ternary complex, the *B* factor of the monomer *A*-bound 2-PG is the lowest (41.9 Å^2^), followed by that of the dimer interface-bound 2-PG (4.3 Å^2^) and lastly the monomer *B*-bound 2-PG (75.6 Å^2^).

Like the interactions of 3-PGA in monomer *A* of the ternary BPGM·3-PGA·2-PG complex, 2-PG at the active site of monomer *A* of the binary complex showed the phosphoryl O atoms making hydrogen-bond interactions with the guanidino groups of Arg10 (3.1 Å), Arg100 (3.1–3.2 Å), Arg116 (2.8–3.0 Å) and Arg117 (3.5 Å), the amide of Asn190 (3.4 Å) and the hydroxyl of Tyr92 (2.7 Å) (Fig. 2 ▸ *d* and Supplementary Fig. S3*a* ). In addition, the phosphoryl O atom forms water-mediated interactions with Asn190, Glu89 and Arg10. The carboxyl group of 2-PG forms hydrogen-bond interactions with the main-chain N atoms of Cys23 (2.6 Å) and Ser24 (2.5–3.5 Å) and the guanidino group of Arg100 (3.5 Å). As noted in monomer *A* of the ternary complex, two water molecules were found at the active site, facilitating the interaction between 2-PG and Arg10, Glu89, Asn190 and Gln251. Unlike 3-PGA, 2-PG does not make a water-mediated interaction with Arg62.

In monomer *B*, the 2-PG phosphoryl O atoms form direct hydrogen-bond interactions with the amide of Asn190 (3.8 Å) and the hydroxyl of Tyr92 (3.3 Å) (Fig. 2 ▸ *e* and Supplementary Fig. S3*b* ). The interactions with Arg116 and Arg117 were missing due to disorder of these residues. Furthermore, the carboxyl of 2-PG makes hydrogen-bond interactions with the main-chain N atoms of Cys23 (2.8 Å) and Ser24 (2.3 Å). However, unlike 2-PG in monomer *B* of the ternary BPGM·3-PGA·2-PG complex, the carboxyl of 2-PG does not form any interaction with Arg100. Also, as observed in monomer *B* of the ternary BPGM·3-PGA·2-PG complex, the structural water molecules were missing. Thus, the water-mediated interactions with His11, Arg10 and Glu89 as seen in monomer *A* of the ternary BPGM·3-PGA·2-PG and binary BPGM·2-PG complexes were not observed.

As in the ternary BPGM·3-PGA·2-PG complex, the active site of monomer *A* of the binary BPGM·2-PG complex is in a closed conformation (Fig. 2 ▸ *b*), while monomer *B*, due to disorder of Arg100, Arg117 and the C-terminus, shows an open conformation (Fig. 2 ▸ *c*). It is clear that both the ternary and binary complex structures exhibit the same pattern of having a disordered monomer *B* while monomer *A* is ordered.

As observed in the ternary BPGM·3-PGA·2-PG complex, we also observed 2-PG bound in the dimer interface of the binary BPGM·2-PG complex, making direct and water-mediated hydrogen-bond interactions with the protein that involve His65 and Glu72 (Figs. 2 ▸ *f*, 2 ▸ *g* and Supplementary Fig. S3*c* ). Specifically, the 2-PG carboxyl group makes a direct hydrogen-bond interaction with the carboxyl of Glu72 (3.5 Å) of monomer *B* and water-mediated hydrogen-bond inter­actions with the imidazole of His65 in monomer *B* and the carboxyl of Glu72 in monomer *A*. The phosphoryl O atoms make direct and water-mediated hydrogen-bond interactions with the imidazole of His65 in monomer *A*, in contrast to the direct hydrogen-bond interaction observed in the ternary BPGM·3-PGA·2-PG complex.

### 3.3. Comparative structural analysis of the ternary BPGM·3-PGA·2-PG, binary BPGM·2PG and previously published BPGM structures

**Figure 3:**
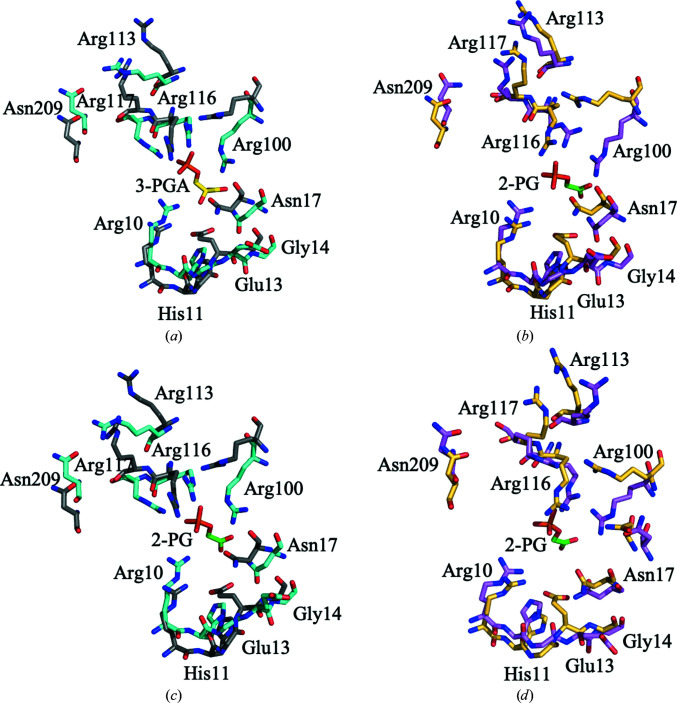
Structural comparison of the ternary BPGM·3-PGA·2-PG, binary BPGM·2-PG and unliganded BPGM (PDB entry 3nfy) structures. (*a*) Superposition of the active site of monomer *A* of BPGM·3-PGA·2-PG (cyan) and PDB entry 3nfy (gray). (*b*) Superposition of the active site of monomer *B* of BPGM·3-PGA·2-PG (pink) and PDB entry 3nfy (yellow). (*c*) Superposition of the active site of monomer *A* of BPGM·2-PG (cyan) and PDB entry 3nfy (gray). (*d*) Superposition of the active site of monomer *B* of BPGM·2-PG (violet) and PDB entry 3nfy (yellow)

Comparison of the ternary BPGM·3-PGA·2-PG or binary BPGM·2PG complex structure with the unliganded BPGM structure (PDB entry 3nfy) shows that ligand binding induces several active-site structural changes in both monomers, although these are more pronounced in monomer *A*. Overall, the main-chain residues from Arg10 to Ser24 at the bottom of the active site move inwards in both monomers *A* and *B* for ligand binding, with Arg10, Asn17, Cys23 or Ser24 making interactions with the bound ligands (Figs. 3 ▸ *a*–3 ▸ *d*). The side chain of Glu13 that occupies the active sites in the unliganded PDB entry 3nfy structure swings outwards to allow ligand binding in the complexes. Several of the residues from Arg100 to Arg117 located at the active-site entrance of BPGM·3-PGA·2-PG and BPGM·2PG show close proximity to the bound ligand, especially in monomer *A* (Figs. 3 ▸ *a*–3 ▸ *d*). For example, the side chains of Arg100, Arg116 and Arg117 of monomer *A* but not monomer *B* make various interactions either with the bound ligand and/or other active-site residues (Fig. 3 ▸). Also, at the surface of the active site of monomer *A* but not monomer *B* or PDB entry 3nfy, Arg113 makes a hydrogen-bond interaction with Asn209 (Fig. 3 ▸). These interactions help to constrain the flexibility of the active-site residues in monomer *A*, fixing it into a closed conformation, which is required for catalysis. The two active sites of PDB entries 2h4x, 2h4z or 2hhj, all with bound ligands, show similar ligand–protein interactions and active-site conformations as monomer *A* in our complexes, while the unliganded active sites in PDB entry 3nfy are similar to monomer *B* of our complexes.

The disorder observed at the C-terminus of BPGM is well documented in the literature due to the dynamic nature and high degree of flexibility of this region. When the C-terminus is resolved, as observed in monomers *A* of the ternary BPGM·3-PGA·2-PG complex and the binary BPGM·2-PG complex, as well as in the previously published structures of BPGM in complex with 2,3-BPG (PDB entry 2h4z) and of BPGM in complex with 3-PGA (PDB entry 2h4x; Wang *et al.*, 2006 ▸), the side chain of Gln251 is observed to be directed towards the center of the active-site pocket, making inter­actions with the bound substrate as well as with Arg100 and Arg116. These interactions help to constrain the flexibility of the Arg100 and Arg116 side chains and fix the active site into a closed conformation. As expected, the absence of the above-described Gln251-mediated interactions with Arg100 and Arg116 in monomers *B* of the ternary BPGM·3-PGA·2-PG and binary BPGM·2-PG complex structures led to significant disorder of the C-termini, resulting in open active-site conformations in these structures. Also as expected, BPGM structures without bound substrate or ligand at the active site, for example the unliganded structure of BPGM (PDB entry 3nfy; Patterson *et al.*, 2010 ▸), have C-terminal disorder with open active-site conformations. A similar observation has been reported for the unphosphorylated/unliganded form of the homologous PGAM1 enzyme, as the last nine residues of the C-terminal tail were found to lack electron density (Wang *et al.*, 2004 ▸). These observations showed that the unphos­phorylated and/or unliganded state of the enzyme exhibits more disordered C-terminal residues than the bound or reacting conformation of the enzyme, in which the C-terminal residues are found to be well defined and covering the active site, maintaining the catalytically active conformation.

Unlike the active sites, it is notable that comparison of the BPGM·3-PGA·2-PG or BPGM·2PG structures with other reported BPGM structures shows no significant structural changes at the dimer interface site even though our structures bind ligand at the dimer interface. Consistent with the above crystallographic observations, when compared with unliganded BPGM (PDB entry 3nfy) using the *DynDom* program to detect domain movements upon ligand binding (Hayward & Berendsen, 1998 ▸), the ternary BPGM·3-PGA·2-PG and binary BPGM·2PG complex structures suggest that the active-site residues 99–118 are involved in domain movement, but no movement is observed in the dimer interface binding site. We speculate that structural changes at the dimer interface could be occurring on ligand binding but are too subtle to be detected at the current resolutions of the structures (2.25 and 2.48 Å). Such structural perturbation in conjunction with ligand binding at the active site of one monomer exerts changes in the dynamic and flexibility of the active site in the second monomer, thus affecting subsequent ligand binding.

### 3.4. Kinetic analysis of the 2-PG activation mechanism

**Figure 4:**
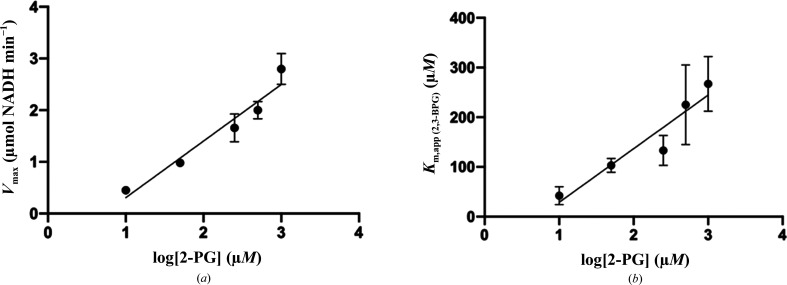
(*a*) The relationship between *V*_max_ and the 2-PG concentration. (*b*) The relationship between the apparent *K*_m_ and the 2-PG concentration.

One of the major functions of BPGM is a 2-PG-stimulated phosphatase activity, which leads to the hydrolysis of 2,3-BPG to 3-PGA. In this study, the rate of 2,3-BPG hydrolysis by BPGM in the presence and absence of 2-PG was measured by coupling the BPGM phosphatase activity to the activity of phosphoglycerate kinase (PGK) and glyceraldehyde-3-phosphate dehydrogenase (GAPDH), and was monitored by following the oxidation of NADH at 340 nm. The apparent kinetic constants were determined for each 2-PG concentration (Table 2 ▸). The results showed that the apparent affinity of 2,3-BPG for BPGM increased on the addition of a 10 µ*M* concentration of 2-PG (*K*_m,app_ = 42 µ*M*) compared with the *K*_m_ value for 2,3-BPG in the absence of 2-PG (*K*_m_ = 77.9 µ*M*) (Table 2 ▸). Interestingly, as the concentration of 2-PG increased beyond 10 µ*M* the apparent affinity of 2,3-BPG for the enzyme starts to decrease linearly from a *K*_m,app_ of 103 µ*M* (at 50 µ*M* concentration of 2-PG) to a *K*_m,app_ of 225.3 µ*M* (at a 500 µ*M* concentration of 2-PG) (Table 2 ▸, Fig. 4 ▸). The *k*_cat_, however, increased linearly with increasing 2-PG concentration (Table 2 ▸, Fig. 4 ▸).

In summary, at a low 2-PG concentration 2,3-BPG seems to bind to the active site with high affinity, with a significant increase in enzyme catalysis, suggesting preferential binding of 2-PG to an allosteric site, possibly the crystallographically observed dimer interface. However, as the concentration of 2-PG increases, an equal increase in the kinetic parameters (*K*_m_ and *k*_cat_) was observed, suggesting binding of 2-PG to the substrate active site. These observations suggest complex enzyme kinetics and require further studies to fully elucidate and/or understand the enzyme mechanism.

## 4. Discussion

The present study reveals structural and kinetic insights into the binding of 2-PG and its activation mechanism of the phosphatase activity of BPGM, which potentially could be leveraged to modulate the activity of BPGM. The activation mechanism of the phosphatase activity by 2-PG and the binding mode of 2-PG to BPGM are poorly understood (Ikura *et al.*, 1976 ▸; Rose & Liebowitz, 1970 ▸; Reynolds, 1986 ▸; Yu *et al.*, 1990 ▸). Pharmacologically, BPGM has long been suggested as a potential target to modulate the 2,3-BPG concentration in the cell, which has been implicated in the pathophysiology of sickle cell anemia (Poillon *et al.*, 1985 ▸, 1995 ▸).

Structural studies of BPGM in complex with 2-PG in the absence and presence of 3-PGA (BPGM·3-PGA·2-PG and BPGM·2-PG complexes) showed not only bound 3-PGA and/or 2-PG at the two active sites but also bound 2-PG at a novel binding site at the dimer interface; the first such report of the latter binding. As previously noted (Patterson *et al.*, 2010 ▸) and observed in our crystallographic studies, Arg100, Arg116 and Arg117 and the C-terminal residues are very dynamic, which appears to be a structural determinant for catalysis. In monomer *A* of both the binary and ternary complexes, these residues are well ordered, resulting in a closed active-site conformation that is similar to that in previously published BPGM structures in complex with 2,3-BPG or 3-PGA (Wang *et al.*, 2006 ▸). However, due to extensive disorder of the side chains of Arg100, Arg116 and Arg117, and the C-terminal residues of monomer *B*, the active site, even though bound with a ligand, assumes a catalytically inactive open conformation similar to the unliganded BPGM structure. As expected, the monomer *B*-bound ligand shows a higher *B* factor, suggesting weak binding. It is noteworthy that the C-terminus is a conserved domain in the homologous phosphoglycerate mutase enzyme (PGAM) family and has been demonstrated to play an important role in the functional activities of the enzymes, including the binding of substrates, the stabilization of the phosphorylated enzyme intermediate and the release of the product by transitioning between the open and closed conformations of the active site (Winn *et al.*, 1981 ▸; Liu *et al.*, 2017 ▸). The deletion of a minimum of seven residues in the C-terminal portion has been shown to abolish the three catalytic activities of BPGM (synthase, phosphatase and mutase; Garel *et al.*, 1989 ▸).

As noted above, none of the previously reported BPGM–ligand complex structures show bound ligand at the dimer interface, and interestingly all show equivalent closed active-site conformations. This is quite different from the observed non-equivalent active sites in both the BPGM·3-PGA·2-PG and BPGM·2-PG complexes that have bound ligand at the dimer interface. We propose the existence of sequential interactions across the BPGM subunits that could potentially be initiated by the binding of 2-PG at the dimer interface, and depending on its concentration, may affect the affinity of BPGM for 2,3-BPG and catalysis.

The existence of the noncatalytic or dimer interface binding site in BPGM, which is possibly allosteric in nature, is in agreement with our kinetic analysis, which suggests that 2-PG increases the affinity of 2,3-BPG and activates the enzyme at low concentration during initial catalysis. It is of note that chloride ion has been demonstrated to activate the phosphatase activity of the PGAM1 enzyme as well as BPGM (Rose & Dube, 1978 ▸; Rose & Liebowitz, 1970 ▸). In PGAM1, chloride was reported to increase the affinity of 2,3-BPG for the enzyme and to be a competitive inhibitor of 2-PG, prompting the authors to postulate that the chloride binding site might be the same as the 2-PG binding site (Rose & Dube, 1978 ▸). In another study, chloride ion was reported to be bound at the dimer interface (near the twofold axis) of PGAM1 (PDB entry 1yfk; Wang *et al.*, 2005 ▸). Based on our study and the literature reports, we propose that 2-PG preferentially binds to the dimer interface. However, as the concentration increases, in addition to the dimer interface, 2-PG binds to the substrate active site.

## 5. Conclusion

Our ternary BPGM·3-PGA·2-PG crystal structure shows a terminal snapshot of the catalytic phosphatase activity, with one subunit undergoing catalysis by tightly binding to the substrate (fully closed conformation), while the other subunit binds 2-PG with a partially closed conformation of the active site in monomer *B*. The binary BPGM·2-PG crystal structure confirmed the ability of 2-PG to bind to the active site and the presence of an alternative noncatalytic binding site at the dimer interface. Until now, there was no clear evidence to suggest that BPGM functions as an allosteric or non-allosteric enzyme. Our findings indicate the existence of a noncatalytic binding site and the possibility of cooperativity and allostery. Nonetheless, further studies are warranted to further support our findings. Identification of the novel binding site at the dimer interface would aid in the design of potential allosteric BPGM modulators, including BPGM phosphatase activators that could potentially reduce 2,3-BPG levels in RBC for the treatment of SCD. Although our study shows some insight into the enzyme activity, further studies are needed to fully elucidate the detailed mechanism.

## Supplementary Material

PDB reference: human bisphosphoglycerate mutase, complex with 3-phosphoglycerate and 2-phosphoglycolate, 7n3r

PDB reference: complex with 2-phosphoglycolate, 7n3s

Raw data for the 2-PG activation kinetics. DOI: 10.1107/S2059798322001802/rr5211sup1.xlsx

Supplementary Figures and Table. DOI: 10.1107/S2059798322001802/rr5211sup2.pdf

## Figures and Tables

**Table 1:** Data-collection and refinement statistics Values in parentheses are for the outer shell.

	BPGM·3-PGA·2-PG	BPGM·2-PG
Data collection		
Wavelength (Å)	1.5418	1.5418
Space group	*P*2_1_2_1_2_1_	*P*2_1_2_1_2_1_
*a*, *b*, *c* (Å)	53.07, 70.86, 159.80	52.64, 70.80, 158.93
α, β, γ (°)	90, 90, 90	90, 90, 90
Resolution range (Å)	27.65–2.25 (2.32–2.25)	29.00–2.48 (2.58–2.48)
Total No. of reflections	229081 (21517)	140609 (15775)
No. of unique reflections	29181 (2677)	21810 (2412)
Completeness (%)	99.20 (100.00)	99.90 (100.00)
Multiplicity	7.90 (8.00)	6.50 (6.50)
〈*I*/σ(*I*)〉	15.0 (4.2)	9.7 (1.8)
*R*_merge_ † (%)	0.084 (0.49)	0.111 (0.71)
Refinement statistics		
Resolution range	27.65–2.25 (2.33–2.25)	29.00–2.48 (2.58–2.48)
Wilson *B* factor (Å^2^)	33.23	45.78
No. of reflections, working set	27539 (2742)	20627 (2527)
No. of reflections, test set	1466 (145)	1093 (135)
Final *R*_cryst_ ‡ (%)	20.3 (32.3)	21.0 (25.8)
Final *R*_free_ § (%)	27.4 (37.3)	28.4 (36.2)
No. of non-H atoms		
Total	4306	4201
Protein	4028	4056
Ligand	29	27
Water	249	118
R.m.s. deviations		
Bond lengths (Å)	0.008	0.008
Angles (°)	0.962	0.988
Average *B* factors (Å^2^)		
Overall	41.78	53.13
Protein	41.76	53.25
Ligand	52.16	60.95
Water	41.01	47.25
Ramachandran plot		
Most favored (%)	96.31	93.51
Allowed (%)	3.48	6.29
Outliers (%)	0.20	0.20
PDB code	7n3r	7n3s

† *R*_merge_ = 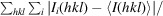 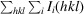 , where *I*(*hkl*) is the integrated intensity of reflection *hkl.*

‡ *R*_cryst_ = 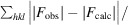 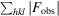 .

§ For *R*_free_ calculations 5% of data were excluded from refinement.

**Table 2:** Enzyme kinetic parameters of 2,3-BPG hydrolysis by the phosphatase activity of BPGM in the presence of 2-PG

[2PG] (µ*M*)	*V*_max_ (µmol NADH min^−1^)	*K*_m,app (2,3-BPG)_ (µ*M*)	k_cat_ (min^−1^)
0	0.20 ± 0.10	77.9 ± 1.7	0.05 ± 0.01
10	0.45 ± 0.06	42 ± 18	0.18 ± 0.02
50	0.98 ± 0.03	102.5 ± 14	0.40 ± 0.01
250	1.66 ± 0.27	133 ± 30	0.66 ± 0.10
500	2.20 ± 0.17	225.3 ± 80	0.88 ± 0.07
