# Supplementary material for: Molecular insight into 2-phosphoglycolate activation of the phosphatase activity of bisphosphoglycerate mutase
Source: Acta Crystallogr D Struct Biol. 2022 Mar 11;78(Pt 4):472–82. doi: 10.1107/S2059798322001802 (PMC8972806; doi:10.1107/S2059798322001802)
Supplement: Supplementary file 2 [file d-78-00472-sup2.pdf]

Acta Cryst D
STRUCTURAL
BIOLOGY

Volume 78 (2022)

Supporting information for article:

Molecular insight into 2-phosphoglycolate activation of bisphosphoglycerate mutase phosphatase activity

Anfal S. Aljahdali, Faik N. Musayev, John W Burgner, Mohini S. Ghatge, Vibha Shekar, Yan Zhang, Abdelsattar M. Omar and Martin K. Safo

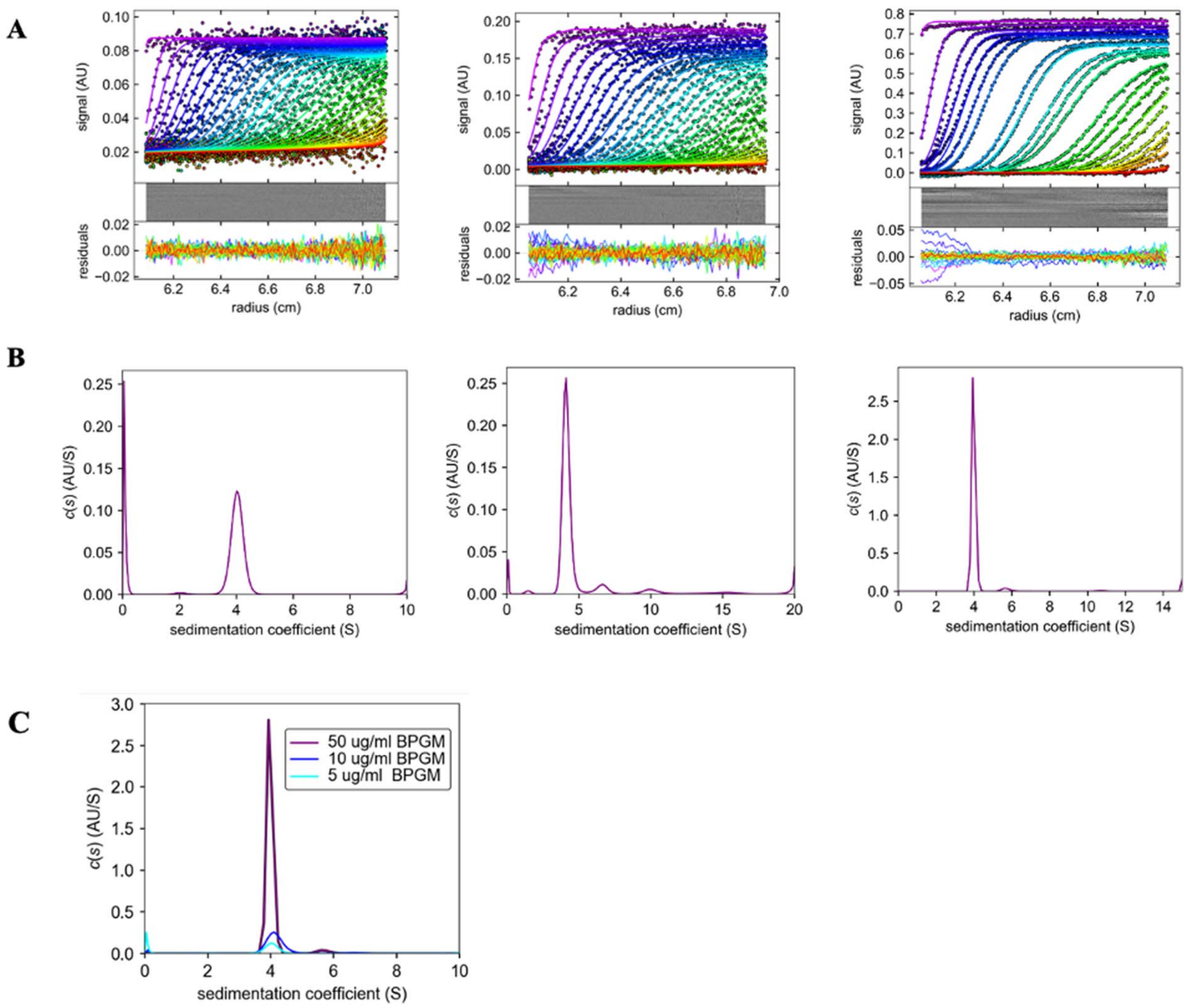

Figure S1 Sedimentation velocity analytical ultracentrifugation analysis of BPGM (**A)** raw sedimentation scan at 230 nm of BPGM at different concentrations 5, 10, 50 ug/ml BPGM (left to right). **(B)** Sedimentation coefficient distribution of BPGM at different concentrations 5, 10, 50 ug/ml BPGM (left to right) **(C)** Sedimentation coefficient distribution of BPGM calculated by SEDFIT for the three SV experiments

Table S1 . Characteristics of the BPGM corrected to pure water

Sample name	$S_{20,w}$, S	Molar Mass	Concentration	Friction ratio
5 ug/ml	4.12S	63.6Kda	0.064 A230	1.309
10 ug/ml	4.23S	62.2KDa	0.159 A230	1.374
50 ug/ml	4.05S	64.0KDa	9.72 A230	1.374
Dimer[a]	4.27S	64.0KDa	0 A 230	N.D.
Monomer	2.91S	32.0KDa	0 A 230	N.D.

[a]Dimer. Values calculated using the program hydropro10 and the X-ray structure factors from the PDB files for dimer and monomer.

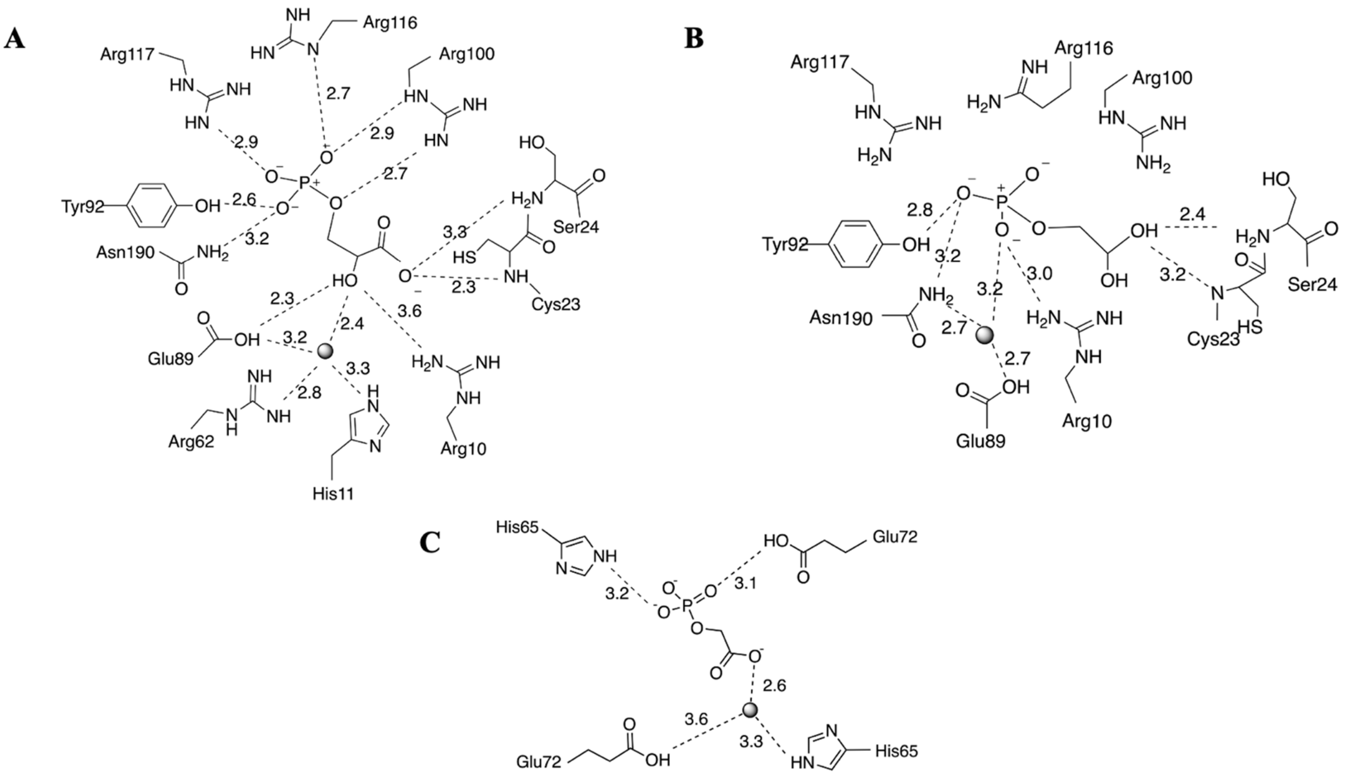

Figure S2 2D schematic representation of ligands interaction in the ternary BPGM•3-PGA•2-PG complex. The interactions are depicted as dashed lines and labeled with bond length (Å). **(A)** interaction of 3-PGA at the active site of monomer A. **(B)** interaction of 2-PG at the active site of monomer B. **(C)** interaction of 2-PG and the dimer interface.

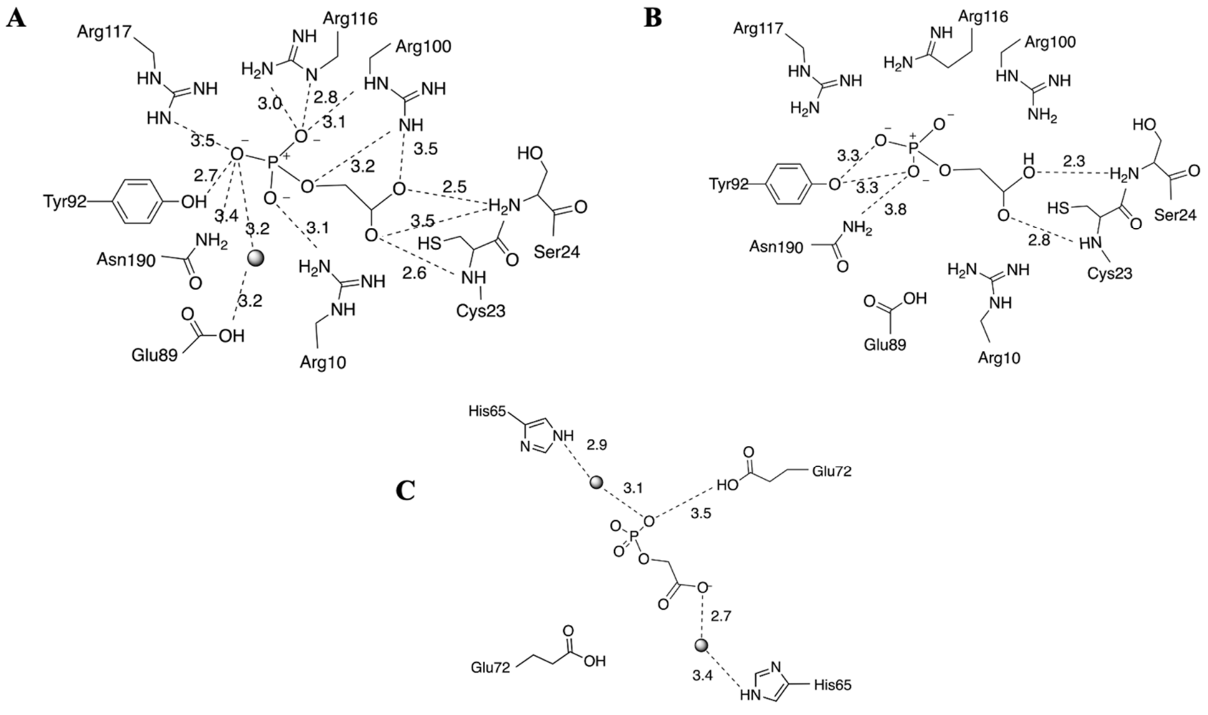

Figure S3 2D schematic representation of ligands interaction in the binary BPGM•2-PG complex. The interactions are depicted as dashed lines and labeled with bond length (Å). **(A)** interaction of 2-PG at the active site of monomer A **(B))** interaction of 2-PG at the active site of monomer B **(C)** interaction of 2-PG at the dimer interface.
